# CD5‐Positive Mature B‐Cell Neoplasm With Large‐Cell Morphology, Predominant Bone Marrow Involvement, and a Leukemic Peripheral Blood Presentation: A Case Report

**DOI:** 10.1155/crh/1476825

**Published:** 2026-10-05

**Authors:** Masamitsu Takaba, Akari Yoda, Shinya Fujisawa

**Affiliations:** ^1^ Department of Hematology Seirei Hamamatsu General Hospital Hamamatsu Japan seirei.or.jp

**Keywords:** BCL6, case report, CD5, monosomy 17, primary bone marrow lymphoma

## Abstract

A 65‐year‐old man presenting with pancytopenia and circulating large atypical cells was initially suspected of having acute leukemia. Flow cytometric analysis demonstrated a mature clonal B‐cell phenotype, and the integrated findings supported a diagnosis of a CD5‐positive mature B‐cell neoplasm with large‐cell morphology, predominant bone marrow involvement, and a leukemic peripheral blood presentation. Based on the available staging data, primary bone marrow DLBCL was suspected but could not be definitively confirmed. The neoplasm was characterized by CD5 expression, a *BCL6* rearrangement, and a complex karyotype including monosomy 17. The patient received six cycles of R‐CHOP followed by two cycles of systemic high‐dose methotrexate as individualized central nervous system prophylaxis. A complete response was confirmed after treatment, with no evidence of recurrence 6 months after treatment completion.

## 1. Introduction

Diffuse large B‐cell lymphoma (DLBCL) is the most common type of malignant lymphoma; however, primary bone marrow lymphoma is rare. Its frequency is estimated to be approximately 1% of malignant lymphomas, and it often follows a rapid clinical course with a poor prognosis [1–3]. In cases with leukemia‐like presentations such as pancytopenia or circulating large atypical cells, differentiation from acute lymphoblastic leukemia or the blastoid variant of mantle cell lymphoma (MCL) is crucial.

Moreover, CD5‐positive DLBCL, which accounts for 5%–10% of DLBCL cases, is known to exhibit a high rate of central nervous system (CNS) relapse [4]. *BCL6* rearrangement has been investigated as a potential prognostic factor in DLBCL; however, the prognostic significance of *BCL6* rearrangement in patients treated with R‐CHOP has been inconsistent [5]. Nevertheless, the clinical characteristics and optimal treatment strategies for primary bone marrow DLBCL with CD5 positivity and complex cytogenetic abnormalities have not yet been established.

Herein, we report a CD5‐positive mature B‐cell neoplasm with large‐cell morphology, predominant bone marrow involvement, and a leukemic peripheral blood presentation. Acute leukemia was initially suspected because of pancytopenia and circulating large atypical cells. Although the integrated cytomorphological, immunophenotypic, and cytogenetic findings strongly suggested primary bone marrow DLBCL, a definitive diagnosis could not be established. The patient was treated with rituximab, cyclophosphamide, doxorubicin, vincristine, and prednisolone (R‐CHOP) followed by systemic high‐dose methotrexate (HD‐MTX), which was selected as individualized CNS prophylaxis based on the overall clinical risk.

## 2. Case Presentation

In September 2025, a 65‐year‐old man with no significant medical history presented to a local clinic with a 2‐week history of fever, general malaise, and loss of appetite. Blood tests revealed pancytopenia and elevated inflammatory markers, prompting referral to Seirei Hamamatsu General Hospital. Laboratory tests showed pancytopenia with 9% large atypical cells in the peripheral blood and a markedly elevated lactate dehydrogenase (LD) level of 1050 U/L (Table 1). Acute leukemia was suspected, and the patient was admitted the same day.

**TABLE 1:** Laboratory findings on admission.

Hematology		Biochemistry		Bone marrow smear	
White blood cell	1490/μL	Total protein	5.9 g/dL	NCC	1.7 × 10^4^/μL
Seg	5.0%	Albumin	3.3 g/dL	Megk	15.0/μL
Band	53.0%	T‐Bilirubin	2.2 mg/dL		
Mono	13.0%	D‐Bilirubin	0.7 mg/dL	Myeloid series	
Lympho	20.0%	AST	86 U/L	Myeloblast	0.0%
Eosino	0.0%	ALT	58 U/L	Promyelo	0.0%
Baso	0.0%	LD	1050 U/L	Myelo	1.6%
Large atypical cells	9.0%	ALP	70 U/L	Meta	2.0%
Red blood cell	263 × 10^4^/μL	γGTP	89 U/L	Band	0.8%
Hemoglobin	9.4 g/dL	BUN	17 mg/dL	Seg	6.2%
Hematocrit	25.8%	Creatinine	1.01 mg/dL	Eosino	0.4%
Platelet	1.2 × 10^4^/μL	Uric acid	7.9 mg/dL	Baso	0.0%
		Na	133 mEq/L	Erythroid series	
		K	4.3 mEq/L	ProE	0.0%
		Cl	96 mEq/L	BasoE	0.0%
Coagulation		Immunology and tumor markers		Poly	1.0%
PT	11.3sec	CRP	9.35 mg/dL	Ortho	0.2%
APTT	32.9sec	IgG	1199 mg/dL	Monocyte	8.8%
Fibrinogen	556.0 mg/dL	IgA	140 mg/dL	Lymphocyte	3.6%
		IgM	27 mg/dL	Plasma cell	0.2%
		Ferritin	3948.0 ng/mL	Large atypical cells	73.2%
		sIL‐2R	5300 U/mL		

*Note:* ALP, alkaline phosphatase; ALT, alanine aminotransferase; APTT, activated partial thromboplastin time; AST, aspartate aminotransferase; Band, band neutrophils; Baso, basophils; BasoE, basophilic erythroblasts; CRP, C‐reactive protein; Eosino, eosinophils; Lympho, lymphocytes; Megk, megakaryocytes; Meta, metamyelocytes; Mono, monocytes; Myelo, myelocytes; Ortho, orthochromatic erythroblasts; Poly, polychromatophilic erythroblasts; ProE, proerythroblasts; Seg, segmented neutrophils; sIL‐2R, soluble interleukin‐2 receptor; γGTP, gamma‐glutamyl transpeptidase.

Abbreviations: BUN, blood urea nitrogen; LD, lactate dehydrogenase; NCC, nucleated cell count; PT, prothrombin time.

Pretreatment bone marrow aspiration revealed that large atypical cells accounted for 73.2% of the nucleated cells and were negative for myeloperoxidase on cytochemical staining. These cells exhibited irregular nuclei with vesicular chromatin, prominent nucleoli, moderate‐to‐abundant basophilic cytoplasm, and cytoplasmic vacuoles (Figure 1A, B). A clot section prepared from the same aspirate contained only scant marrow tissue and was insufficient for adequate histopathological evaluation. Consequently, the pattern of neoplastic infiltration and tissue‐based immunophenotypic characteristics could not be assessed. A bone marrow trephine biopsy was not performed. Contrast‐enhanced computed tomography performed before corticosteroid treatment showed no lymphadenopathy, mass lesions, or hepatosplenomegaly. Although acute leukemia was initially suspected, preliminary flow cytometric analysis of the bone marrow indicated a mature clonal B‐cell neoplasm. Flow cytometry, conventional cytogenetic analysis, and FISH were performed using the pretreatment bone marrow aspirate. Because the disease was rapidly progressive, prednisolone was initiated as bridging therapy after contrast‐enhanced computed tomography (CT) and preliminary flow cytometric evaluation, while the cytogenetic and FISH results were pending. This bridging therapy led to an improvement in the patient’s general condition and gradual decreases in LD and C‐reactive protein levels. Complete lymphoma staging, including pretreatment fluorodeoxyglucose positron emission tomography/CT (FDG‐PET/CT), could not be performed before corticosteroid treatment. A diagnostic lumbar puncture was not performed, and no dedicated baseline CNS assessment was undertaken. Flow cytometric analysis of the pretreatment bone marrow aspirate identified a discrete abnormal cell population accounting for 38.4% of the analyzed events. The difference between the morphologic and flow cytometric percentages may reflect methodological differences, including different denominators, hemodilution, sampling differences between aliquots, cell loss during processing, and gating strategy. The abnormal population was identified on the basis of its combined CD45, forward‐scatter, and side‐scatter characteristics. Compared with residual lymphocytes, the abnormal cells showed relatively dim CD45 expression, increased forward scatter, and low‐to‐intermediate side scatter. They expressed CD19, CD20, CD22, and CD5 and demonstrated surface kappa light‐chain restriction, whereas CD10, CD34, terminal deoxynucleotidyl transferase (TdT), and cytoplasmic CD3 were not expressed. The expression of mature B‐cell antigens and surface light‐chain restriction, together with the absence of CD34 and TdT, supported the diagnosis of a mature clonal B‐cell neoplasm and argued against B‐lymphoblastic leukemia/lymphoma (Figure 1C). The tested leukemia‐associated fusion transcripts were not detected; however, this finding was ancillary and was not used as a primary basis for distinguishing the neoplasm from B‐lymphoblastic leukemia/lymphoma. The soluble interleukin‐2 receptor level was elevated at 5300 U/mL, although this finding was nonspecific.

**FIGURE 1:**
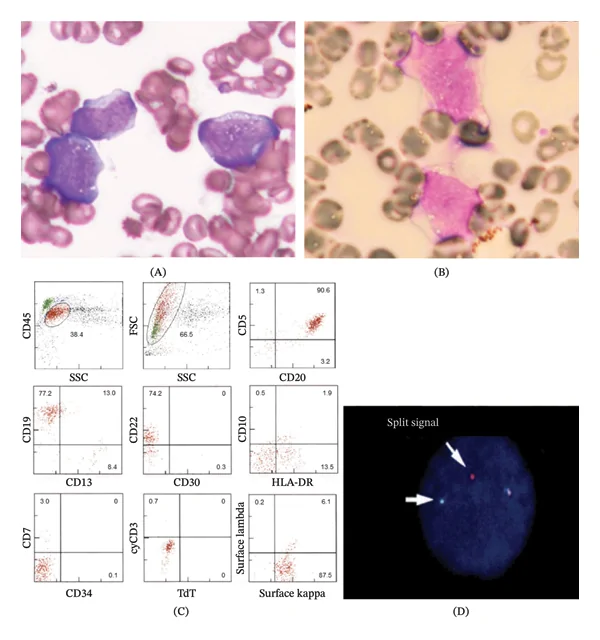
Bone marrow findings at the time of initial diagnosis. (A) May–Giemsa staining of the bone marrow smear. A marked proliferation of large atypical cells with moderate‐to‐abundant basophilic cytoplasm and prominent nucleoli is observed (original magnification, × 500). (B) MPO cytochemical staining of the bone marrow aspirate showed that the large atypical cells were negative (original magnification, × 500). (C) Flow cytometric analysis of the pretreatment bone marrow aspirate. A discrete abnormal cell population accounting for 38.4% of all analyzed events was identified on the basis of its combined CD45, forward‐scatter, and side‐scatter characteristics. Compared with residual lymphocytes, the abnormal cells showed relatively dim CD45 expression, increased forward scatter, and low‐to‐intermediate side scatter. The gated abnormal cells expressed CD19, CD20, CD22, and CD5 and demonstrated surface kappa light‐chain restriction, whereas CD10, CD34, terminal deoxynucleotidyl transferase, and cytoplasmic CD3 were not expressed. The complete flow cytometry panel included CD1a, CD2, CD3, cytoplasmic CD3, CD4, CD5, CD7, CD8, CD10, CD13, CD14, CD19, CD20, CD22, CD30, CD33, CD34, CD38, CD41, CD45, CD56, CD117, CD138, HLA‐DR, glycophorin A, MPO, TdT, and surface kappa and lambda light chains. Representative diagnostically relevant plots are shown. (D) Pretreatment fluorescence in situ hybridization analysis using a *BCL6* break‐apart probe. Split signals indicating a *BCL6* rearrangement were observed in 69 of 100 analyzed nuclei (69%; laboratory‐established positivity cutoff, > 2%). FSC, forward scatter; MPO, myeloperoxidase; SSC, side scatter; TdT, terminal deoxynucleotidyl transferase.

R‐CHOP therapy was subsequently initiated for the suspected aggressive mature B‐cell neoplasm. Following treatment initiation, symptoms resolved quickly; after the myelosuppression phase, hematopoiesis recovered, and the patient became transfusion‐independent. No serious complications, such as tumor lysis syndrome, were observed. Conventional G‐banding analysis of the pretreatment bone marrow demonstrated the following complex karyotype: 48,XY,del(1)(p?),t(3;22)(q27;q11.2),add(5)(q31),add(8)(q13),add(10)(q22),−12,del(13)(q?),−17,+18,−20,+mar1,+mar2,+mar3[20/20]. The same abnormal clone was detected in all 20 metaphases analyzed, with no normal metaphases or cytogenetically distinct subclones identified. The karyotype was classified as complex because it contained multiple independent numerical and structural chromosomal abnormalities. The finding of −17 represented loss of one entire chromosome 17, that is, monosomy 17. FISH analysis performed at SRL, Inc. using a dual‐color break‐apart probe flanking the *BCL6* locus at 3q27 demonstrated a split‐signal pattern in 69 of 100 nuclei (69%; laboratory‐established positivity cutoff, > 2%). FISH analyses for *CCND1::IGH*, *MYC*, and *BCL2* rearrangements were negative. The integrated clinical, cytomorphological, immunophenotypic, and cytogenetic findings strongly suggested primary bone marrow DLBCL, although a definitive diagnosis could not be established. The disease was clinically regarded as Stage IV based on the documented bone marrow involvement. When provisionally assessed using the NCCN‐IPI for DLBCL, the patient had a score of 6 (age, 2 points; LD, 2 points; Stage IV disease, 1 point; and bone marrow involvement as a high‐risk extranodal site, 1 point), placing him in the high‐risk group.

The clinical course is shown in Figure 2. After three cycles of R‐CHOP therapy, large atypical cells were no longer observed morphologically in the bone marrow, and no abnormal cell population was detected by flow cytometry. Conventional G‐banding analysis no longer identified the previously detected abnormal karyotypic clone, including monosomy 17. *BCL6* break‐apart FISH showed no split signals in the 100 nuclei evaluated (0%). *TP53* deletion FISH, which had not been performed before treatment, was also performed after three cycles of R‐CHOP using a *TP53* locus probe and a chromosome 17 pericentromeric control probe. No deletion signal pattern was identified among the 100 nuclei evaluated (0%; laboratory‐established positivity cutoff, > 2%). Bone marrow histological assessment was not performed after three cycles of R‐CHOP. After completing a total of six cycles of R‐CHOP therapy, two cycles of HD‐MTX therapy were administered as individualized CNS prophylaxis. Repeat conventional G‐banding analysis after treatment completion showed no detectable abnormal karyotypic clone, and repeat *BCL6* break‐apart and *TP53* deletion FISH analyses were also negative (0/100 nuclei for each). Bone marrow examination, including morphologic assessment and flow cytometry, and FDG‐PET/CT after treatment completion showed no evidence of residual disease, and a complete response was confirmed. At the latest follow‐up, 12 months after diagnosis and 6 months after completion of treatment, no clinical, hematologic, or radiologic evidence of recurrence was observed. Given the limited follow‐up period, longer observation is required to assess the durability of the response.

**FIGURE 2:**
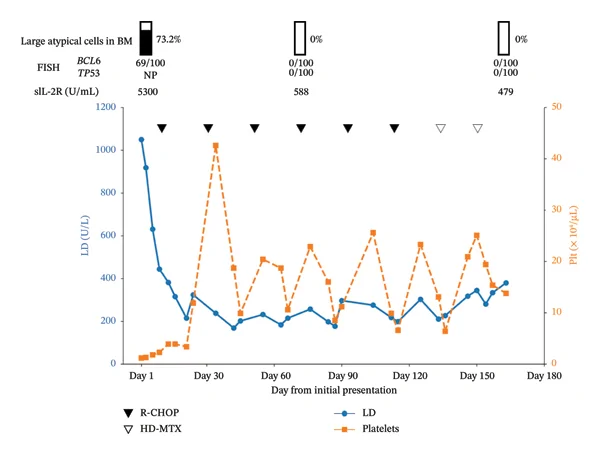
Clinical course of the patient. The patient received six cycles of R‐CHOP therapy (▼), followed by two cycles of HD‐MTX administered as individualized central nervous system prophylaxis (▽). The line graph shows changes in LD levels and platelet counts according to the number of days after the initial presentation. Serum sIL‐2R levels, the percentage of large atypical cells in bone marrow smears, and the results of *BCL6* break‐apart and *TP53* deletion FISH analyses are shown at the corresponding time points. After three cycles of R‐CHOP, large atypical cells were no longer detected in the bone marrow, and the *BCL6* rearrangement was no longer detected. *TP53* deletion FISH, which was first performed at this time point, was negative. After completion of six cycles of R‐CHOP and two cycles of HD‐MTX, repeat *BCL6* break‐apart and *TP53* deletion FISH analyses remained negative, and a complete response was confirmed by bone marrow examination and FDG‐PET/CT. BM, bone marrow; FDG‐PET/CT, fluorodeoxyglucose positron emission tomography/computed tomography; FISH, fluorescence in situ hybridization; HD‐MTX, high‐dose methotrexate; LD, lactate dehydrogenase; NP, not performed; Plt, platelet count; R‐CHOP, rituximab, cyclophosphamide, doxorubicin, vincristine, and prednisolone; sIL‐2R, soluble interleukin‐2 receptor.

## 3. Discussion

This case involved a CD5‐positive mature B‐cell neoplasm with large‐cell morphology, predominant bone marrow involvement, and a leukemic peripheral blood presentation. Primary bone marrow lymphoma is a rare entity, accounting for approximately 1% of malignant lymphomas [1–3]. Primary bone marrow lymphomas often present with cytopenias, whereas circulating lymphoma cells are less frequently observed. In cases with a leukemic peripheral blood presentation, careful differentiation from B‐lymphoblastic leukemia/lymphoma and the blastoid variant of MCL is essential.

Bone marrow cytomorphology and flow cytometry were central to the diagnostic evaluation in this case. The large atypical cells exhibited irregular nuclei with vesicular chromatin, prominent nucleoli, moderate‐to‐abundant basophilic cytoplasm, and cytoplasmic vacuoles, findings compatible with a large B‐cell neoplasm with an immunoblastic appearance. Flow cytometry demonstrated mature B‐cell antigen expression, surface kappa light‐chain restriction, CD5 positivity, CD10 negativity, and absence of CD34 and TdT, supporting the diagnosis of a mature clonal B‐cell neoplasm and arguing against B‐lymphoblastic leukemia/lymphoma.

Cytogenetic and FISH analyses further characterized the neoplasm but were not independently diagnostic. The absence of *CCND1::IGH* and *BCL2* rearrangements made MCL and follicular lymphoma, respectively, less likely, although neither could be completely excluded. Cyclin D1‐negative MCL may harbor *CCND2* or *CCND3* abnormalities, and SOX11 immunohistochemistry would have been informative; however, no bone marrow core biopsy was obtained, and the available clot section contained insufficient tissue for reliable assessment. Similarly, follicular lymphoma or transformation from an underlying follicular lymphoma may occur in the absence of a *BCL2* rearrangement. Other aggressive mature B‐cell neoplasms, including high‐grade B‐cell lymphoma, intravascular large B‐cell lymphoma, EBV‐positive DLBCL, primary splenic DLBCL with bone marrow involvement, and transformation from an underlying small B‐cell lymphoma, could not be completely excluded.

Integrated cytomorphological, immunophenotypic, and cytogenetic findings were highly suggestive of DLBCL with predominant bone marrow involvement and a leukemic peripheral blood presentation. Published operational criteria for primary bone marrow lymphoma require pathologically confirmed bone marrow involvement, regardless of peripheral blood involvement, and the absence of lymph node, splenic, hepatic, osseous, or other extramedullary disease [3, 6]. Thus, the presence of 9% circulating tumor cells was not incompatible with these criteria. Although contrast‐enhanced CT showed no lymphadenopathy, mass lesions, or hepatosplenomegaly, adequate histopathological confirmation was unavailable, and pretreatment FDG‐PET/CT was not performed. These limitations precluded adequate assessment of the marrow infiltration pattern and tissue‐based immunophenotype and complete exclusion of low‐volume extramedullary disease. Therefore, definitive classification as primary bone marrow DLBCL or DLBCL, NOS with bone marrow and peripheral blood involvement could not be established, and we used a descriptive diagnostic designation based on the available findings.

Compared with the previously reported series summarized in Table 2, the present case shared several features of primary bone marrow lymphoma, including older age, fever, cytopenias, elevated LD, and predominant bone marrow involvement [1–3, 7]. Leukemic peripheral blood involvement was relatively uncommon, whereas CD5 expression itself has been reported previously [1–3, 7]. This combination of CD5 expression, *BCL6* rearrangement, a complex karyotype including monosomy 17, and leukemic peripheral blood involvement was not reported in the cited series. Thus, the co‐occurrence of these clinical, immunophenotypic, and cytogenetic features represents the principal distinguishing feature of this case.

**TABLE 2:** Comparison of the present case with published series of primary bone marrow lymphoma.

Feature	Chang et al.[1]	Bhagat et al.[2]	Wang et al.[3]	Kimbrough et al.[7]	Present case
No. of patients	12	4	66	2	1
Age/sex	Median 62.5 years (44–80); M/F, 5/7	Median 52 years (51–64); M/F, 1/3	Median 60 years (29–81); M/F, 42/24	71/F; 75/F	65/M
B symptoms	9/12	NR	26/47^∗^	2/2^∗∗^	Positive (fever)
Cytopenia	Anemia, 11/12; thrombocytopenia, 9/12 Leukopenia, 3/12; pancytopenia, 2/12	Anemia, 4/4; thrombocytopenia, 3/4, Leukopenia, 2/4	Grades 1–2, 18/34^∗^, Grades 3–4, 16/34^∗^	Anemia, 1/2; thrombocytopenia, 1/2, Leukopenia, 1/2	Pancytopenia
Circulating lymphoma cells	1/12	0/4	NR	1/2	Present (9% atypical large cells)
LD elevation	10/12	NR	30/38^∗^	2/2	Elevated (1050 U/L)
Marrow involvement pattern	Not systematically reported; intrasinusoidal in 2/12	Interstitial, 4/4; paratrabecular component, 1/4	NR	Case 1, diffuse; Case 2, NR	Not assessable (no core biopsy)
CD5 expression and COO phenotype	CD5+, 4/5 evaluated; COO, NR	CD5, NR; GCB, 4/4 by modified Hans algorithm	CD5+, 4/28 evaluated; COO, NR	CD5+, 0/2; non‐GCB, 1/1 evaluated (COO NR in 1)	CD5+; COO, not assessable
Cytogenetic and molecular findings	NR	NR	NR	Case 1, NR; Case 2, complex karyotype with MYC and BCL2 rearrangements	BCL6 rearrangement; complex karyotype including monosomy 17
Initial treatment	Supportive care, *n* = 4 CHOP/COP, *n* = 4; R‐CHOP/R‐COP, *n* = 4	R‐CHOP, n = 2 Other chemotherapy, *n* = 2	R‐CHOP/CHOP‐based, *n* = 23 Intensive regimens, *n* = 5	Case 1, R‐CHOP + HD‐MTX Case 2, R‐CHOP ⟶ DA‐EPOCH‐R + IT‐MTX	R‐CHOP + HD‐MTX
Response	CR, 4/4	Remission, 2/4	CR, 29/57; PR, 9/57	Case 1, CR; second CR after salvage therapy Case 2, residual disease	Early CR
Follow‐up/outcome	Follow‐up, 1–56 months; alive, 4/12	Follow‐up, 6 months–15 years; died, 2/4; relapsed, 2/4	Median survival, 19 months	Case 1, relapse at 3 years; alive 4 months after salvage therapy Case 2, CNS progression at 3 months; died	CR maintained 6 months after treatment completion

*Note:* CHOP, cyclophosphamide, doxorubicin, vincristine, and prednisone; COP, cyclophosphamide, vincristine, and prednisone; R‐CHOP, rituximab plus CHOP; R‐COP, rituximab plus COP; HD‐MTX, high‐dose methotrexate; DA‐EPOCH‐R, dose‐adjusted etoposide, prednisone, vincristine, cyclophosphamide, doxorubicin, and rituximab; IT‐MTX, intrathecal methotrexate; GCB, germinal center B‐cell‐like.

Abbreviations: CNS, central nervous system; COO, cell of origin; CR, complete response; F, female; LD, lactate dehydrogenase; M, male; NR, not reported; PR, partial response.

^∗^Of 66 patients, 47 were included in the retrospective analysis; denominators vary according to data availability.

^∗∗^Case 1 had weight loss attributed to poor appetite; formal B‐symptom criteria were not reported.

A prospective single‐arm phase II study reported favorable outcomes with DA‐EPOCH‐R combined with HD‐MTX in patients with newly diagnosed CD5‐positive DLBCL, although this regimen was not directly compared with R‐CHOP [8]. In the present case, the large atypical cells in the bone marrow and the abnormal population detected by flow cytometry were no longer detectable after three cycles of R‐CHOP, before HD‐MTX was administered. This temporal sequence indicates that the early systemic response had already been achieved before HD‐MTX administration and is consistent with sensitivity of the neoplasm to R‐CHOP. HD‐MTX was subsequently administered as individualized CNS prophylaxis rather than as treatment for persistent systemic disease. Although the reason for the favorable initial response cannot be determined from a single case, longer follow‐up is required to assess the durability of the response.

Although outcomes in primary bone marrow DLBCL vary widely and no standard treatment has been established [2, 6, 7, 9], CD5‐positive DLBCL is known to carry a significantly higher risk of CNS relapse than CD5‐negative DLBCL [4]. Furthermore, DLBCL with predominant bone marrow involvement and circulating tumor cells, as observed in the present case, has also been suggested to be associated with an increased risk of CNS relapse [7]. Although the benefit of systemic HD‐MTX for CNS prophylaxis in DLBCL remains controversial, CNS relapse in CD5‐positive DLBCL frequently involves the brain parenchyma, where intrathecal prophylaxis may be insufficient [4]. In the present case, systemic HD‐MTX was selected as individualized CNS prophylaxis after careful consideration of the CD5‐positive phenotype, predominant bone marrow involvement, circulating tumor cells, and overall clinical risk.

In addition to CD5 positivity, the neoplasm harbored a *BCL6* rearrangement and a complex karyotype including monosomy 17. Although *BCL6* rearrangement has been associated with adverse outcomes in some studies, its prognostic significance in patients with DLBCL treated with R‐CHOP has been inconsistent and may depend on other clinical and molecular factors [5]. Because *TP53* is located at 17p13.1, monosomy 17 would be expected to result in loss of one *TP53* allele. However, the mutation status of the remaining allele was not assessed, and the available findings therefore did not establish biallelic or multihit *TP53* alteration or the presence of a gain‐of‐function *TP53* mutation. After three cycles of R‐CHOP, the abnormal karyotypic clone, including monosomy 17, and the *BCL6* rearrangement were no longer detected, and *TP53* deletion FISH was negative. Repeat G‐banding and *BCL6* and *TP53* FISH after treatment completion also showed no detectable abnormalities. However, because pretreatment *TP53* FISH and mutation analysis were not performed, the pretreatment *TP53* status could not be fully characterized.

Additional limitations included incomplete pretreatment *TP53* assessment and the relatively short follow‐up period.

In conclusion, this case describes a CD5‐positive mature B‐cell neoplasm with large‐cell morphology, predominant bone marrow involvement, and a leukemic peripheral blood presentation, characterized by a *BCL6* rearrangement and a complex karyotype including monosomy 17. Integrated cytomorphological, flow cytometric, cytogenetic, and FISH findings strongly suggested primary bone marrow DLBCL, although definitive classification was not possible. The patient showed an early favorable response to R‐CHOP, followed by individualized HD‐MTX CNS prophylaxis, but longer follow‐up is required to assess the durability of disease control. This case also highlights that large atypical cells in the peripheral blood or bone marrow of a patient with pancytopenia should not automatically be interpreted as leukemic blasts. Early integration of cytomorphology with flow cytometry and appropriate ancillary studies is important in distinguishing acute leukemia from aggressive mature lymphoid neoplasms.

## Author Contributions

M.T. contributed to the conception of the case report, acquisition and interpretation of clinical data, and drafting of the manuscript. A.Y. contributed to the acquisition and interpretation of clinical data and critically reviewed the manuscript. S.F. supervised the work and critically reviewed the manuscript. Masamitsu Takaba had full access to all of the data in this study and takes complete responsibility for the integrity of the data and the accuracy of the data analysis.

## Funding

This study did not receive any specific funding.

## Disclosure

All authors have read and approved the final version of the manuscript.

## Consent

Written informed consent was obtained from the patient for publication of this case report, including relevant clinical details and accompanying images.

## Conflicts of Interest

The authors declare no conflicts of interest.

## Data Availability

The data that support the findings of this case report are available from the corresponding author upon reasonable request. The data are not publicly available due to privacy and ethical restrictions.
